# Retrospective validation of a machine learning clinical decision support tool for myocardial infarction risk stratification

**DOI:** 10.1049/htl2.12017

**Published:** 2021-08-31

**Authors:** Saarang Panchavati, Carson Lam, Nicole S. Zelin, Emily Pellegrini, Gina Barnes, Jana Hoffman, Anurag Garikipati, Jacob Calvert, Qingqing Mao, Ritankar Das

**Affiliations:** ^1^ Division of Data Science Dascena, Inc. Houston Texas USA

## Abstract

Diagnosis and appropriate intervention for myocardial infarction (MI) are time‐sensitive but rely on clinical measures that can be progressive and initially inconclusive, underscoring the need for an accurate and early predictor of MI to support diagnostic and clinical management decisions. The objective of this study was to develop a machine learning algorithm (MLA) to predict MI diagnosis based on electronic health record data (EHR) readily available during Emergency Department assessment. An MLA was developed using retrospective patient data. The MLA used patient data as they became available in the first 3 h of care to predict MI diagnosis (defined by International Classification of Diseases, 10th revision code) at any time during the encounter. The MLA obtained an area under the receiver operating characteristic curve of 0.87, sensitivity of 87% and specificity of 70%, outperforming the comparator scoring systems TIMI and GRACE on all metrics. An MLA can synthesize complex EHR data to serve as a clinically relevant risk stratification tool for MI.

## 1 BACKGROUND

In the United States, over 6.5 million patients annually are evaluated for chest pain in the Emergency Department (ED); in these instances, myocardial infarction (MI) is a key diagnostic consideration [1]. MI is defined as an acute myocardial injury indicated by elevated serum biomarkers of myocardial necrosis (typically cardiac troponins) with clinical evidence of acute myocardial ischemia [2]. 14% of people who suffer an MI in the United States die as a result [3]. Those who survive may experience significant morbidity and are at elevated risk for recurrent MI and death [4, 5]. Treatment outcomes are highly dependent on time‐sensitive diagnosis and intervention, which aims to restore blood flow to ischemic myocardium to prevent or minimise tissue damage and death [6]. Indeed, the mortality risk is greatest in the earliest stages of an acute MI, underscoring the importance of rapid and accurate detection [5].

The diagnosis of MI is based on suggestive clinical signs and symptoms, electrocardiogram (ECG) abnormalities, and elevated cardiac troponins; cardiac imaging and stress tests may further assist in evaluation [7]. However, the diagnostic process is complicated by symptom variability across patient populations and overlap with the symptoms of other syndromes [8, 9, 10]. Up to one‐third of MI are silent, or occurring without symptoms [11]. ECG abnormalities may be absent, progressive, or non‐specific in the context of prior cardiac events and underlying ischemic disease [12]. While newer generation troponin tests and high sensitivity assays detect troponin elevation with great sensitivity even in the initial hours after symptom onset [13, 14], elevations are also detected in a range of other cardiac and non‐cardiac conditions. MI encompasses both ST‐elevation MI (STEMI) and Non‐ST‐elevation MI (NSTEMI). STEMI represent more severe ischemic events and are ideally recognised early in patient assessment based on the pathognomonic ST elevation on ECG. However, ED crowding has been identified as a potential challenge to delivering high quality care, including the timeliness of assessment [15, 16]. The ECG abnormalities in NSTEMI are variable and may be progressive, and NSTEMI cannot be distinguished from the related but less severe condition of unstable angina without evaluating cardiac troponins [9]. Patients, thus, may be boarded in the ED for prolonged periods and receive serial ECGs as well as troponin measurements at 3 or 6 h intervals [9]. ED length of stay may also be extended for patients with atypical symptom presentations during diagnostic work‐up for MI and differential diagnoses [16]. Thus, enhanced risk stratification at 3 h represents a clinically relevant timeframe.

Diagnostic accuracy is also largely dependent on the diagnosing clinician, introducing further variability into the diagnostic process [17, 18, 19]. Amidst this complexity, it is perhaps unsurprising that the individual components of the standard clinical evaluation demonstrate limited accuracy for diagnosing MI, most notably in terms of sensitivity [20]. Clinical prediction tools, such as the thrombolysis in myocardial infarction (TIMI) score, may be used to help rule out MI or to determine the types of treatments appropriate for a patient's estimated level of risk [20]. However, the uptake and routine use of these tools are constrained by their inherent interruption of the clinical workflow by relying on physicians to tabulate scores at the bedside or on a computer [21].

Delays in appropriate treatment are associated with increased mortality risks [9] and unnecessary treatment for MI can introduce treatment‐related harms to improperly diagnosed patients [22] or lower‐risk patients who could be treated with less intensive, guideline‐recommended pharmacotherapies. These harms highlight the opportunity for innovative approaches to support MI diagnosis and treatment planning which are accurate, easy to integrate into the clinical workflow, and can be utilised within the initial hours of a patient's assessment within the ED.

Machine learning (ML) approaches to the diagnosis and prediction of MI have been leveraged in a growing body of research, the preponderance of which focus on risk stratification or outcomes predictions following an MI [23, 24]. ML approaches to assist with specific steps in the initial diagnostic process have also been investigated, including approaches to improving ECG interpretation, identifying misplacement of ECG leads, and enhancing cardiac imaging capabilities to detect acute MI [25, 26, 27]. However, a Machine Learning Algorithm (MLA) based clinical decision support (CDS) tool that supports rapid rule in or rule out of MI, and provides actionable estimations of risk to guide the intensity of interventions, would improve care by minimising delays to individualised, risk‐appropriate treatment. Ideally, such a tool would use data routinely available in the electronic health record (EHR) and would not require additional physician inputs so as not to impede the clinical workflow. Towards this end, we have developed a novel MLA that can predict MI using only data available within the first 3 h of a patient's hospital‐based assessment, and which does not require serial troponins or repeated ECG.

## 2 METHODS

### 2.1 Data processing

Patient data collected between 2011 and 2015 at a large academic medical center in the Western United States were used in this study. Data were extracted from patients admitted to any hospital ward and included patient demographics, past medical history, vital signs, and laboratory results. Data were collected passively and de‐identified in compliance with the Health Insurance Portability and Accountability Act (HIPAA).

For the purposes of this study, data was included from patients with at least one of each of the following measurements in the first 3 h of the patient encounter: systolic blood pressure, diastolic blood pressure, respiratory rate, peripheral oxygen saturation and troponin I. The requirement for a troponin measurement prior to the time point for algorithm deployment was included to ensure selection of a high‐risk patient population in which MI was under diagnostic consideration. The information extracted by the MLA from the EHR to compute scores are presented in Table 1. Beyond a troponin measurement and the minimum vital sign measurements, no other features were explicitly required by the MLA to generate a prediction score, in order to maximise utility of the algorithm in live clinical environments in which different data may be available for different patients at the time of prediction generation.

**TABLE 1:** Structured data extracted from the electronic health record if available in the patent record, used by the machine learning algorithm to predict myocardial infarction diagnosis

Demographics	
Age	Sex
**History of present illness**	
Chest pain	
**Past medical history**	
Prior myocardial infarction	Diabetes mellitus
Hypertension	Hyperlipidemia
Tobacco use	
**Vital signs**	
Systolic blood pressure	Diastolic blood pressure
Heart rate	Respiratory rate
Peripheral oxygen saturation (SpO_2_)	Temperature
**Laboratory values**	
Sodium	Troponin I
Potassium	Lactate
Blood urea nitrogen	Hematocrit
Creatinine	Platelet count
Bicarbonate	White blood cell count
Glucose	International normalised ratio (INR)
Aspartate transaminase	Blood pH
Alanine transaminase	Urine output
Total bilirubin	

The algorithm was designed to generate a score 3 h after the start of the patient encounter. Input features were added as they became available at a refresh rate of 10 min. If there were no new measurements after 10 min, measurements were carried forward. For timepoints at which a given measurement had not been collected yet, a null value was reported and the null value was implicitly handled by the ML classifier as an input.

### 2.2 Gold standard

Encounters were considered positive for MI if an International Classification of Diseases (ICD), 10th revision (ICD‐10) code for MI was listed for the encounter. The following ICD‐10 codes were used to identify MI: I21.0, I21.1, I21.2, I21.3, I21.4, I22.0, I22.1, I22.2, I22.8, I22.9. ICD codes were used to define the positive class based on prior literature demonstrating that the codes perform with acceptably high accuracy as proxies for MI diagnosis [28, 29, 30, 31, 32, 33, 34]. ICD codes have been shown to perform with high sensitivity, specificity, and positive predictive values in identifying MI within hospitalisation databases [28, 29, 30]. All patient encounters not labelled with one of the specified ICD‐10 codes were considered negative. Whereas the MLA was only allowed to access to EHR data available within the first 3 h of an encounter for MI predictions, the gold standard could be established at any point during a patient's ED assessment or subsequent hospitalisation.

### 2.3 Comparison to standard of care

The diagnostic evaluation of patients with suspected MI is composed of findings taken from the history of present illness, past medical history, physical exam, and diagnostic tests. Validated risk stratification tools may be used to integrate findings from these different sources to assist clinicians in predicting the likelihood that a given patient will experience an MI or associated major adverse outcomes. For this study, we chose to compare our MLA to two popular risk stratification tools for MI, the TIMI score and Global Registry of Acute Coronary Events (GRACE) score [34, 35, 36]. Based on clinical measurements taken at initial ED presentation, the GRACE score has been used to prognosticate outcomes such as MI and mortality during hospital admissions and periods of up to 3 years following admission [14]. The TIMI score also uses initial ED observation to prognosticate adverse outcomes related to cardiovascular morbidity. Both GRACE and TIMI scores are recommended by clinical societies to guide treatment planning decisions in the context of possible MI [14, 37]. Since this MLA was designed to serve as an alternative prognostication method in high risk patients, these common, guideline‐recommended risk scores used to predict adverse cardiovascular outcomes were selected as comparators.

GRACE and TIMI translate key clinical findings into a final predictive score by weighting the findings and accumulating the numerical weights. Based on commonly cited cutoff points above which patients are no longer low risk for an MI and may be considered to be at elevated risk for an MI, the clinical operating points were defined as ≥2 for TIMI and ≥108 for GRACE [9, 38, 39]. The data necessary to tabulate TIMI and GRACE were not available for each unique encounter. To remedy this, we used a previously reported method to impute missing data which has also been used by other MLAs [40, 41]. ECG data were not available in this dataset and were thus not included in calculations of any TIMI or GRACE score; this impacted the maxima for each score. As the MLA produces probability scores ranging from 0 to 1, TIMI and GRACE scores were scaled into probability scores to facilitate comparison by dividing the score by the respective model's maximum achievable score within our dataset (5.3 for TIMI, 305 for GRACE). Scaled scores were then plotted on receiver operating characteristic (ROC) curves. The original TIMI and GRACE risk scores inputs are provided in Supplementary Table 1. Data inputs used to tabulate the adjusted TIMI and GRACE are provided in Supplementary Table 2.

### 2.4 Machine learning algorithm

A novel MLA was developed to predict MI, trained using the inputs in Table 1. The MLA is a gradient boosted tree model implemented with the XGBoost (XGB) library in Python [42, 43]. The XGB method uses collections of gradient‐boosted decision trees to classify data. For example, a patient's creatinine level may place a patient along one of two paths. Using this example of creatinine, if this measurement is not available, the MLA would select a branching direction that results in the MLA making a better prediction on average. Multiple creatinine branching points may exist on a single decision tree with, for example, one that follows a male branching point and one that follows the female branching point, allowing two cutoff values for creatinine that are conditioned on the gender of the patient to exist. The end of the decision tree has one “leaf” that represents each patient encounter, with the patients in each leaf predicted to have the same probability of the outcome. The final score is then the sum of all trees. XGB models progressively incorporate any new splits in the branching points along the range of the values of its inputs, incorporating that information into new branches and new trees. Further, this training adjusts for the addition of any new component and how it may impact this component's ability to reduce the loss function versus the contribution to the model's general complexity. The objective or cost function, or the “loss function,” quantifies each new branch's ability to improve the training accuracy after new branches are added to the model, and also to the model's complexity and overfitting. Thus, weaker decision tree base learners adjust rapidly and effectively from large amounts of data, and learn even from missing data when using XGB. The XGB method was chosen for this study due to its simplicity, high performance, and useful implementation features, which provides options for handling imbalanced classes and regularisation [42, 44]. This model takes the first 3 h of data from Table 1 as input features, as data is made available, separated by 10 min intervals. As per the gold standard, patient encounters were labelled uniquely positive or negative, with encounters with MI defined as the positive class and those without as the negative class.

The model was trained and tested using an 80:20 train:test split. To train the model, 80% of patients were randomly selected and the remaining 20% were used as a hold‐out set to test generalisation after training. A stratified threefold cross‐validation grid search [45] was conducted for hyperparameter optimisation. The training set was split into 3 separate folds and each combination of hyperparameters was used to train the model on two folds and validate on the remaining one. The combination of hyperparameters that resulted in the highest validation AUC was saved as the optimal hyperparameters, which were then used to train the entire training set. Optimal hyperparameters included learning rate, regularisation penalty, positive weight scaling, and maximum tree depth. Final optimised hyperparameters for XGB were learning rate of 0.05, regularisation penalty of 3, positive weight scaling of 1, and maximum tree depth of 3.

Performance metrics are reported as the performance of the model on the testing set. Operating points for TIMI and GRACE were defined using the cutoff points described above. The operating point selected for the MLA along its ROC curve was selected to maximise sensitivity and specificity relative to the comparator models. Model performance at this operating point was compared to comparators’ performance at their respective operating points in terms of sensitivity, specificity, likelihood ratios and diagnostic odds ratios.

## 3 RESULTS

**FIGURE 1:**
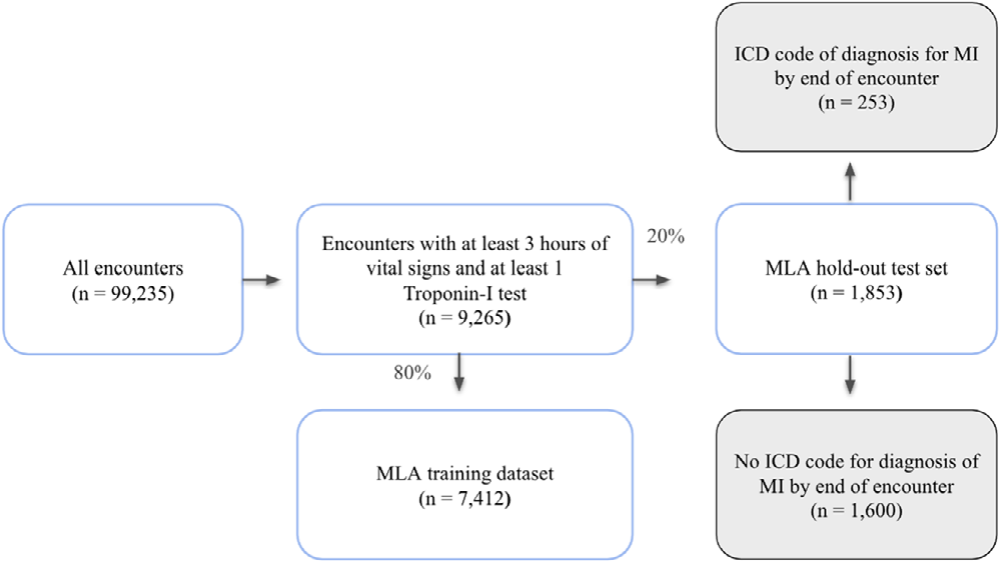
Patient encounters used to train and test a machine learning algorithm to predict myocardial infarction based on electronic health data available within the first 3 h.

We analysed 99,235 patient encounters, of which 9,265 encounters had at least 3 h of vital sign data recorded and at least one troponin‐I test ordered during the encounter. The hold‐out test set consisted of 1,853 patient encounters, of which 253 encounters were positive for MI per the gold standard (Figure 1).

All data presented below pertain to the hold out validation dataset used to test the MLA. Fisher's exact test was used to evaluate statistical significance of demographic differences between patients with and without MI, with a significance level of *p* < 0.05. Patients who experienced an MI were less likely to be young and less likely to be female (Table 2). Significant differences in past medical history were noted between patients in the positive and negative classes, with MI patients more likely to have diabetes, hypertension, dyslipidemia, peripheral vascular disease, angina, heart failure, chronic kidney disease, and chronic obstructive pulmonary disease. Patients diagnosed with an MI were also more likely to have a history of prior MI. The median age for the MI population was 72 years (interquartile range (IQR): 60, 81), compared to 70 years (IQR: 58, 82) in the population without MI. Demographic data for the complete dataset used for training and testing is presented in Supplementary Table 3.

**TABLE 2:** Demographic information for the hold out test dataset used to test the machine learning algorithm

	Patients with MI (*n* = 253)	Patients without MI (*n* = 1,600)	*p*‐values
**Age (years)**			
<30	1 (0.4%)	41 (2.6%)	0.04
30–49	25 (9.9%)	160 (10.0%)	1.00
50–59	34 (13.4%)	223 (13.9%)	0.92
60–69	53 (20.9%)	362 (22.6%)	0.63
70–79	55 (21.7%)	341 (21.3%)	0.87
<80	85 (33.6%)	473 (29.6%)	0.21
**Sex**			
Male	169 (66.8%)	831 (51.9%)	0.01
Female	84 (33.2%)	769 (48.1%)	0.01
Unknown	0 (0.0%)	0 (0.0%)	1.0
**Race**			
American Indian or Alaska Native	0 (0.0%)	0 (0.0%)	1.0
Asian	58 (22.9%)	379 (23.7%)	0.87
Black or African American	23 (9.1%)	254 (15.9%)	0.004
Native Hawaiian or Other Pacific Islander	9 (3.6%)	33 (2.1%)	0.17
White or Caucasian	116 (45.8%)	703 (43.9%)	0.59
Other	43 (17.0%)	211 (13.2%)	0.11
Unknown/declined	4 (1.6%)	20 (1.2%)	0.56
**Ethnicity**			
Hispanic or Latino	16 (6.3%)	140 (8.8%)	0.61
**Comorbid conditions**			
Obesity	13 (5%)	143 (9%)	0.05
Diabetes mellitus	108 (43%)	516 (32%)	0.001
Dyslipidemia	130 (51%)	560 (35%)	< 0.001
Hypertension	201 (79%)	1126 (70%)	0.003
Peripheral vascular disease	28 (11%)	69 (4%)	< 0.001
Angina	52 (21%)	96 (6%)	< 0.001
Heart failure	116 (46%)	473 (30%)	< 0.001
CKD	100 (40%)	422 (26%)	< 0.001
HIV infection and AIDS	7 (3%)	51 (3%)	0.85
Dementia	25 (10%)	173 (11%)	0.74
COPD	35 (14%)	314 (20%)	0.03
Depression	24 (9%)	214 (13%)	0.10
Current tobacco use	25 (10%)	174 (11%)	0.74
Prior MI	61 (24%)	148 (9%)	< 0.001
Prior ischemic stroke or TIA	2 (1%)	20 (1%)	0.76

Abbreviations: Acquired immunodeficiency syndrome (AIDS); chronic kidney disease (CKD); chronic obstructive pulmonary disease (COPD); human immunodeficiency virus (HIV); myocardial infarction (MI); transient ischemic attack (TIA).

**FIGURE 2:**
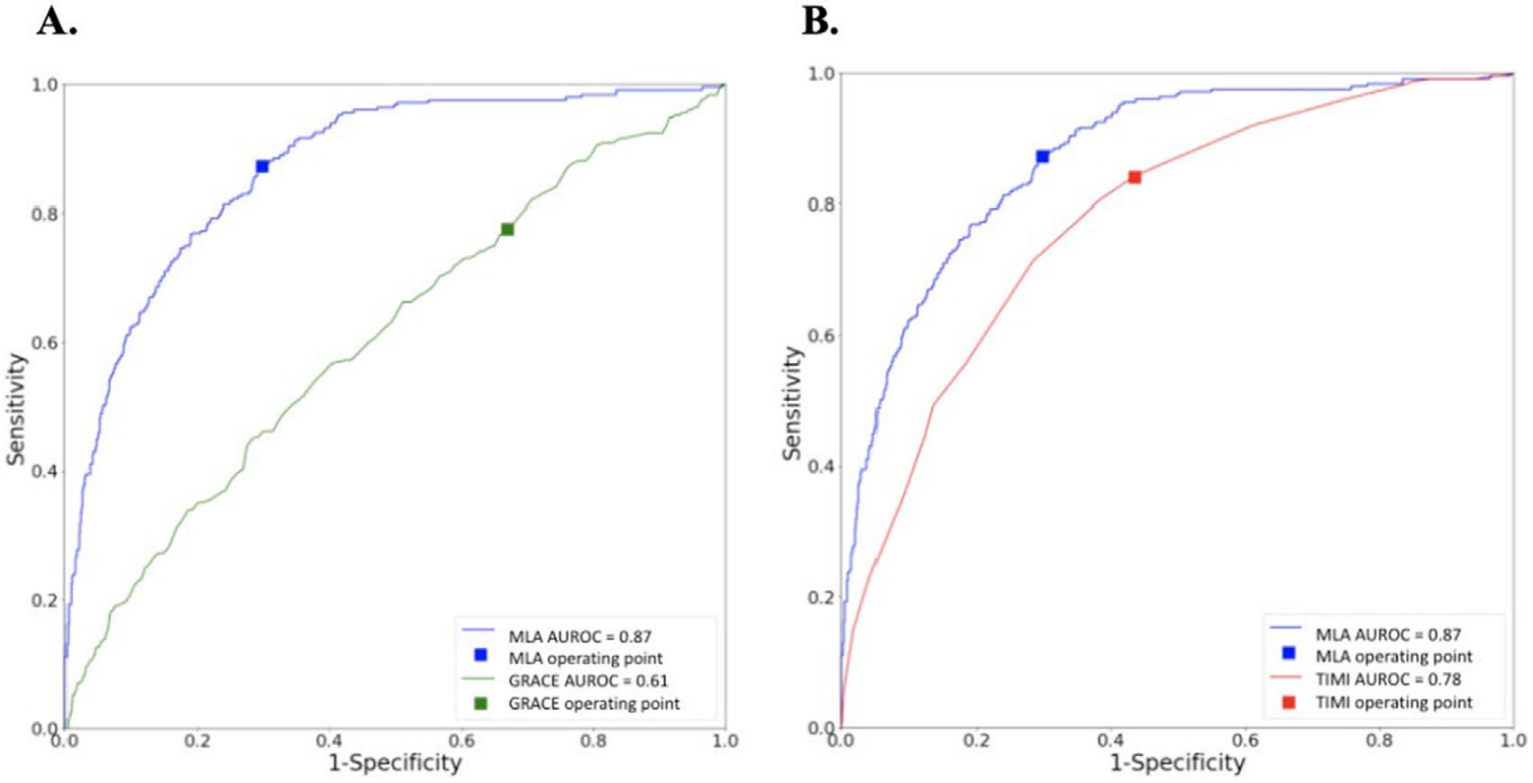
Area under receiving operating characteristic curves and clinical operating points for (A) machine learning and GRACE clinical prediction model of myocardial infarction diagnosis and (B) machine learning and TIMI clinical prediction model of myocardial infarction diagnosis.

The MLA's ability to predict MI was assessed on the hold out test dataset and compared to the adjusted TIMI and GRACE scores. ROC curves were plotted (Figure 2), with the MLA demonstrating superior performance in classifying cases compared to the clinical prediction rules.

The MLA outperformed the comparator tools in predicting MI on all metrics evaluated (Table 3). The MLA achieved an area under the receiver operating characteristic curve (AUROC) of 0.87, sensitivity of 0.87 and specificity of 0.70. The TIMI achieved an AUROC of 0.78 with a sensitivity of 0.84 and specificity of 0.57, performing better than the GRACE. GRACE demonstrated the lowest AUROC, sensitivity and specificity of the three models at 0.61, 0.78 and 0.33, respectively.

**TABLE 3:** Performance metrics of machine learning algorithm and comparator models for myocardial infarction prediction

	MLA	GRACE	TIMI
**AUROC**	0.87	0.61	0.78
**Sensitivity**	0.87	0.78	0.84
**Specificity**	0.70	0.33	0.57
**LR+**	3.0	1.2	1.9
**LR‐**	0.18	0.67	0.28
**DOR**	16.5	1.8	7.0
**PPV**	0.32	0.16	0.24
**NPV**	0.97	0.91	0.96

Abbreviations: Area under the receiver operating characteristic (AUROC); likelihood ratio (LR); machine learning algorithm (MLA).; diagnostic odds ratio (DOR); positive predictive value (PPV); negative predictive value (NPV).

**FIGURE 3:**
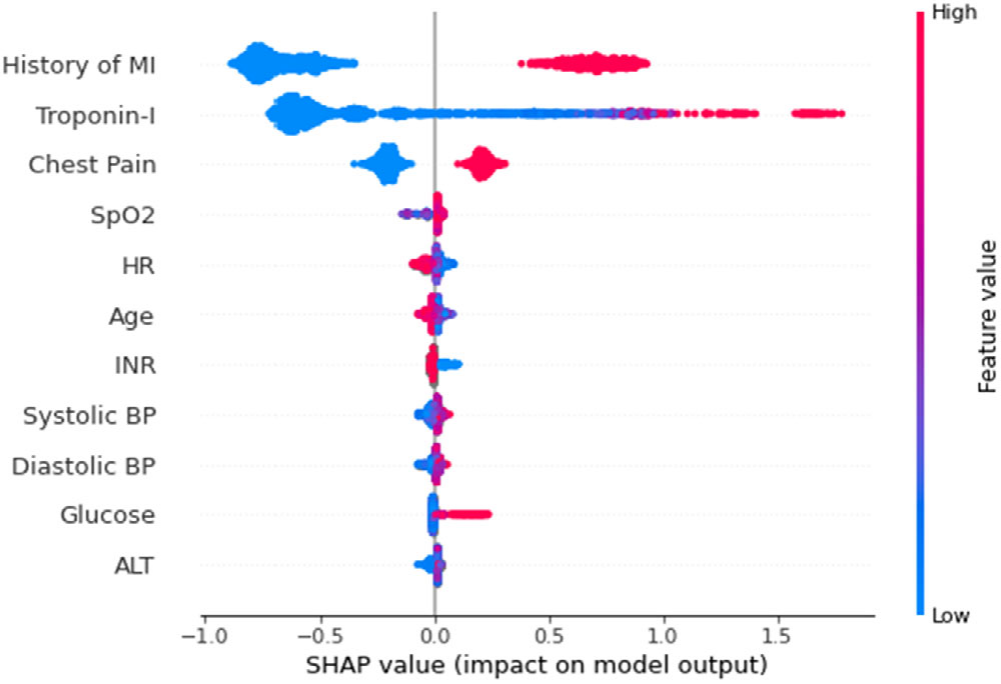
Top unique feature correlations and distribution of feature importance for each patient encounter for machine learning models. Input variables are ranked in descending order of feature importance. Red indicates a high feature value and blue indicates a low feature value. Points to the right and left sides of the line of neutral contribution resulted in higher and lower prediction scores, respectively. Abbreviations: alanine aminotransferase (ALT); blood pressure (BP); heart rate (HR); international normalised ratio (INR); myocardial infarction (MI); peripheral oxygen saturation (SpO2).

Feature correlations and distribution of feature importance for MLA performance was evaluated using a SHAP summary plot (Figure 3). Prior MI, troponin I values, and chest pain were among the most important EHR features for predicting MI. As expected, Troponin I values are positively correlated with MI. Rather than a single threshold for high versus low however, the model has multiple thresholds for troponin I conditioned on the patient's other features. These thresholds can differ by branch or tree. In general, troponin I above the thresholds result in increases in the final score whereas troponin I below the thresholds result in decreases in the final score.

## 4 DISCUSSION

Delays in appropriate treatment for MI are associated with increased mortality risks [9]. Conversely, unnecessary treatment (or unnecessarily aggressive treatment) can introduce treatment‐related harms to improperly diagnosed patients [46], or lower‐risk patients who could be treated with less intensive, guideline‐recommended pharmacotherapies. These harms highlight the opportunity for innovative approaches to support MI risk stratification tools that are accurate, easy to integrate into the clinical workflow, and can be utilised within the initial hours of a patient's assessment within the ED. In this work, we describe such a tool to support risk‐appropriate medical intervention through timely and accurate risk stratification for MI among high‐risk patients. Using the EHR database of a large academic medical center, an MLA was developed which could extract readily available data from EHRs as they were updated without interrupting the clinical workflow. The MLA was developed to predict MI with high accuracy based on available inputs and outperformed clinical prediction tools which are currently suggested for risk stratification in clinical guidelines produced by the American Heart Association, American College of Cardiology [37] and the European Society of Cardiology [14].

The results of Table 3 demonstrate that the MLA performed substantially better than the TIMI and GRACE scores in predicting MI at any point during a patient encounter, achieving an AUROC of 0.87 compared to 0.78 for TIMI and 0.61 for GRACE. Notably, the tool was both more sensitive and more specific than the comparator risk stratification tools (Table 3; Supplementary Table 4). The MLA also achieved greater PPV and NPV than either GRACE or TIMI, indicating that it was more accurate in truly identifying high risk cases where MI ultimately occurred and correctly ruling out low risk cases in which MI did not occur. The MLA, thus, performed with high accuracy in predicting an eventual diagnosis of MI, using only the data available within an initial 3 h assessment window (Table 1). Unlike the more complex GRACE and TIMI scoring systems, which incorporate features that are not routinely assessed across patients (e.g. Killip class, 1 week history of aspirin use), the MLA is capable of making predictions using only routinely collected patient data, and therefore does not require additional work on the part of the clinician. Further, an MLA provides flexibility, as the operating point and/or threshold for alerts can be adjusted to reflect clinician feedback and to maximise the clinical utility of the tool to meet the needs of individual hospitals.

In addition to comparing performance to GRACE and TIMI, feature importance for all MLA inputs was assessed using SHAP values, which consider the contributions of each feature in making predictions (Figure 3). Past medical history of MI, Troponin I values, and chest pain were among the most important features for the MLA's performance. Given the substantive research on chest pain as a cardinal symptom of MI, elevated troponin as a defining aspect of MI, and prior MI as a risk factor for recurrent MI, the MLA identified relevant relationships in the data consistent with the published literature [3, 15, 39].

The gold standard encompassed ICD‐10 codes for both STEMI and NSTEMI, and was thus designed to predict both STEMI and NSTEMI. As described in the introduction, STEMI represent more severe ischemic events and are ideally recognised early in patient assessment, making enhanced risk stratification at 3 h a clinically relevant timeframe. Within the complete training and test dataset, an elevated troponin result was returned at a median of 2 h and mean of 7 h into the patient encounter. While this rough proxy for time of clinical diagnosis does not take into account the time at which a clinician may first recognise suggestive ECG abnormalities, it does underscore the length of the diagnostic window for MI in a real‐world setting and the utility of a CDS risk stratification tool which can facilitate hospitalisation and clinical management decisions within this window.

In the growing body of research on MLAs as CDS tools, these tools have performed with high sensitivity and specificity using variable types of input data [21]. However, many studies have been limited by training and testing in small sample populations, and some have required exhaustive inputs or additional manual data entry to make a prediction [21]. The MLA described in this work was trained and tested using a large dataset (*n* = 9,265) and minimal exclusion criteria were applied to maximise the generalisability of findings. Our MLA can extract and integrate multiple features from the EHR into predictions; however, beyond a single troponin measurement and at least one measurement for four routinely measured vital signs, the MLA does not require that any other individual feature be present in order to make a prediction. Previous researchers have suggested that incorporating troponin measurements into MLAs may enhance algorithms’ predictive ability [17]. In 2019, Than et al. reported on the prospective performance of the myocardial‐ischemic‐injury‐index (MI^3^), a gradient boosted algorithm which uses patient demographics and two sequential high‐sensitivity cardiac troponin values to predict likelihood of MI diagnosis [47]. MI^3^ demonstrated high sensitivity and specificity and was effective in ruling out patients without MI, with a NPV of 99.7% [47]. However, MI^3^ cannot make predictions without serial troponin measurements, which may not be readily available in an early assessment window in all clinical practice contexts [47]. The MLA we have developed can incorporate repeated troponin measurements as input features, but is not inhibited by their absence from making a prediction.

This work has several limitations. First, patient data were collected from a cohort of patients at a single academic medical center, which limits generalisability. Second, there was a higher percentage of male patients as compared to female patients among patients with MI in the hold out validation dataset. This sex‐based difference may reflect existing bias in the diagnostic process for MI; recent research has suggested that lower troponin thresholds may be appropriate for diagnosing MI in women [12]. Third, while ICD codes for MI have been demonstrated to perform with high sensitivity, specificity, and positive predictive value for identifying MI in health records [28, 29, 30], it is possible that some patient encounters were not properly classified. In particular, some recent research in the era of high‐sensitivity troponin tests has demonstrated a lack of concordance between ICD 10 labels and a clinical diagnosis of MI per the 4th Universal Definition of MI [48]. However, other studies using historical data have demonstrated acceptable concordance (e.g. kappa statistic *K* > 0.6) between ICD codes for MI and clinician adjudicated diagnosis [31, 34]. As the data used in this study were collected in a comparable historical time period (2011–2015), ICD codes applied during this time period can be considered a reasonable proxy for clinical diagnosis. Fourth, the absence of ECG data in this dataset represents a limitation on the use of TIMI and GRACE as comparator models, as both scores incorporate ECG findings as inputs. The performance of the adjusted TIMI (AUROC = 0.78) and GRACE (AUROC = 0.61) in this study are largely consistent with previous research on unadjusted TIMI and GRACE scores to predict MI and other major adverse cardiovascular events in ED patients [49, 50, 51]. A further limitation on the use of the GRACE and TIMI scores as comparators is that neither tool was explicitly designed to only predict MI diagnosis within a hospital stay. However, both scores have been used to prognosticate cardiovascular adverse outcomes, such as MI, among patients at high risk of acute coronary syndrome, such that these clinical risk scores remain the most appropriate comparators for this novel MLA. Fifth, as this was a retrospective dataset provided via contract for research purposes, constraints on the breadth of data provided were present. For example, while the dataset indicated the timing of troponin test result, troponin test result in ng/mL, and whether a result was abnormal, no information on the type(s) or exact troponin assays used over the time period of the study were available. Finally, as this study was conducted retrospectively, future research on the prospective performance of this algorithm is warranted to support its utility as a CDS tool. In future work, this work will be extended by prospective assessment across data derived from different hospital settings, and investigate the use of sex‐specific troponin cut‐offs to improve diagnostic performance and utility of the tool in clinical practice.

## 5 CONCLUSIONS

We have developed an MLA that can risk stratify patients for MI with high accuracy. Troponin remains an important input for the MLA, similar to standard MI clinical diagnostic and risk stratification tools, and future research directions may explore serial troponin as an input for this model in order to assess the impact on predictive accuracy. However, while our current MLA can incorporate repeated troponin measurements, only a single troponin measurement is required in order to make a prediction. As this MLA performs with high sensitivity and specificity, we propose that the use of a risk stratification MLA may support clinical management and hospitalisation decisions early in the diagnostic process.

## Declarations

**Ethics Approval and Consent to Participate**: Data were collected passively and de‐identified in compliance with the Health Insurance Portability and Accountability Act (HIPAA). Since data were de‐identified and collected retrospectively, this study was considered non‐human subjects research and did not require Institutional Review Board approval.

## LIST OF ABBREVIATIONS

AUROC: Area under the receiver operating characteristic curve
CDS: Clinical decision support
DOR: Diagnostic odds ratio
ECG: Electrocardiogram
EHR: Electronic health record
ED: Emergency Department
GRACE: Global Registry of Acute Coronary Events
HIPAA: Health Insurance Portability and Accountability Act
ICD‐10: International classification of diseases, 10th revision
IQR: Interquartile range
LR: Likelihood ratio
MLA: Machine learning algorithm
MI: Myocardial infarction
MI^3^
: Myocardial‐ischemic‐injury‐index
NPV: Negative predictive value
NSTEMI: Non‐ST‐elevation myocardial infarction
PPV: Positive predictive value
ROC: Receiver operating characteristic
STEMI: ST‐elevation myocardial infarction
TIMI: Thrombolysis in Myocardial Infarction

## Supporting information

SUPPORTING INFORMATION
