# Supplementary material for: Retrospective validation of a machine learning clinical decision support tool for myocardial infarction risk stratification
Source: Healthc Technol Lett. 2021 Aug 31;8(6):139–47. doi: 10.1049/htl2.12017 (PMC8667565; doi:10.1049/htl2.12017)
Supplement: Supplementary file 1 — SUPPORTING INFORMATION [file HTL2-8-139-s001.docx]

**Supplementary Table 1.** Original data inputs for TIMI and GRACE risk scores.

| **TIMI** (13) | **GRACE** (35) |
| --- | --- |
| - Age 65 years or older - Prior coronary stenosis of 50% or more - Three or more risk factors for coronary artery disease - Use of aspirin in the preceding 7 days - Two or more anginal events in the past 24 hours - ST-segment deviation on electrocardiogram at presentation - Elevated cardiac biomarkers | - Age - Heart rate - Systolic blood pressure - Serum creatinine - Killip class - Cardiac arrest at admission - Elevated cardiac markers - ST-segment deviation |

**Supplementary Table 2.** Data inputs used to tabulate adjusted TIMI and GRACE scores for this study.

| **TIMI** | **GRACE** |
| --- | --- |
| - Age 65 years or older - Known coronary artery disease - Risk factors for heart disease, which include:   - high blood pressure (greater than 140/90)   - smoking (being a current smoker)   - low HDL cholesterol (less than 40 mg/dL)   - Diabetes mellitus - Angina as a presenting symptom - Elevated serum cardiac biomarkers | - Age - Heart rate - Systolic blood pressure - Serum creatinine - History of congestive heart failure - Diagnosis of cardiac arrest on admission - Elevated cardiac biomarkers |

**Supplementary Table 3.** Demographic information for the complete dataset used to train and test the machine learning algorithm to predict MI. Fisher’s exact test was used to determine significance at p < 0.05. *Abbreviations: Acquired Immunodeficiency Syndrome (AIDS); Chronic kidney disease (CKD); Chronic obstructive pulmonary disease (COPD); Human Immunodeficiency Virus (HIV); Myocardial infarction (MI).*

|  | Patients with MI  (N=1,265) | Patients without MI  (N =8000) | p-value |
| --- | --- | --- | --- |
| **Age (years)** | | | |
| < 30 | 7 (0.6%) | 182 (2.3%) | < 0.001 |
| 30 - 49 | 122 (9.6%) | 919 (11.5%) | 0.06 |
| 50 - 59 | 192 (15.2%) | 1263 (15.8%) | 0.62 |
| 60 - 69 | 293 (23.2%) | 1794 (22.4%) | 0.56 |
| 70 - 79 | 248 (19.6%) | 1542 (19.3%) | 0.79 |
| ⩾ 80 | 403 (31.9%) | 2300 (28.8%) | 0.03 |
| **Sex** | | | |
| Male | 789 (62.4%) | 4175 (52.2%) | < 0.001 |
| Female | 475 (37.5%) | 3824 (47.8%) | < 0.001 |
| Unknown | 1 (0.1%) | 1 (0.0%) | 1.0 |
| **Race** | | | |
| American Indian or Alaska Native | 5 (0.4%) | 21 (0.3%) | 0.39 |
| Asian | 296 (23.4%) | 2010 (25.1%) | 0.20 |
| Black or African American | 164 (13.0%) | 1294 (16.2%) | 0.003 |
| Native Hawaiian or Other Pacific Islander | 41 (3.2%) | 184 (2.3%) | 0.05 |
| White or Caucasian | 532 (42.1%) | 3360 (42.0%) | 0.98 |
| Other | 206 (16.3%) | 1040 (13.0%) | 0.002 |
| Unknown/Declined | 21 (1.7%) | 91 (1.1%) | 0.13 |
| **Ethnicity** | | | |
| Hispanic | 100 (7.9%) | 676 (8.4%) | 0.55 |
| **Comorbid conditions** | | | |
| Diabetes | 509 (40%) | 2566 (32%) | < 0.001 |
| Hypertension | 1010 (80%) | 5539 (69%) | < 0.001 |
| Dyslipidemia | 594 (47%) | 2790 (35%) | < 0.001 |
| Peripheral vascular disease | 113 (9%) | 341 (4%) | < 0.001 |
| Angina | 261 (21%) | 532 (7%) | < 0.001 |
| Heart failure | 584 (46%) | 2440 (30%) | < 0.001 |
| CKD | 485 (38%) | 2217 (27%) | < 0.001 |
| HIV infection and AIDS | 45 (4%) | 245 (3%) | 0.34 |
| Dementia | 110 (9%) | 842 (11%) | .05 |
| Obesity | 102 (8%) | 723 (9%) | .29 |
| COPD | 197 (16%) | 1494 (19%) | 0.008 |
| Depression | 133 (11%) | 949 (12%) | 0.17 |
| Current Tobacco Use | 165 (13%) | 817 (10%) | 0.003 |
| Prior MI | 278 (22%) | 717 (9%) | < 0.001 |
| Prior ischemic stroke or transient ischemic attack | 11 (1%) | 129 (2%) | 0.05 |

**Supplementary Table 4.** Performance metrics of machine learning algorithm and comparator models for myocardial infarction prediction. Metrics for MLA are listed for two operating points; operating point one was selected to maximize sensitivity and specificity relative to the comparator models, operating point two was selected to have comparable specificity relative to the TIMI score. *Abbreviations: Area under the receiver operating characteristic (AUROC); likelihood ratio (LR); machine learning algorithm (MLA); diagnostic odds ratio (DOR); positive predictive value (PPV); negative predictive value (NPV).*

|  | **MLA**  operating point 1 | **MLA**  operating point 2 | **TIMI** | **GRACE** |
| --- | --- | --- | --- | --- |
| **AUROC** | 0.87 | 0.87 | 0.78 | 0.61 |
| **Sensitivity** | 0.87 | 0.93 | 0.84 | 0.78 |
| **Specificity** | 0.70 | 0.61 | 0.57 | 0.33 |
| **LR+** | 3.0 | 2.4 | 1.9 | 1.2 |
| **LR-** | 0.18 | 0.11 | 0.28 | 0.67 |
| **DOR** | 16.5 | 21.5 | 7.0 | 1.8 |
| **PPV** | 0.32 | 0.273 | 0.24 | 0.16 |
| **NPV** | 0.97 | 0.983 | 0.96 | 0.91 |
